# Supplementary material for: Are carbon emissions trading and green financial instruments synergistic? -Comprehensive quantitative research based on content analysis
Source: PLoS One. 2024 Mar 7;19(3):e0298601. doi: 10.1371/journal.pone.0298601 (PMC10919601; doi:10.1371/journal.pone.0298601)
Supplement: S1 Data — (ZIP) [file pone.0298601.s001.zip › Data/地级市Z_F工作报告.zip!/╦─┤¿/╥╦▒÷╩╨/└┤╫╘╣1⁄2╓┌║┼í╛┬φ┐╦╩2╛▌═°í┐_2016.docx]

各位代表：

我代表市人民政府，向大会报告工作，请各位代表审查，并请市政协委员提出意见。

“十二五”及2015年工作回顾

“十二五”时期是经济发展进入新常态、各项工作充满挑战的五年，也是全市上下攻坚克难、经济社会发展取得显著成效的五年。五年来，我们认真贯彻落实党中央国务院、省委省政府的决策部署，在市委的坚强领导下，在市人大及其常委会的法律监督、工作监督和市政协的民主监督下，深入实施“2365”发展战略，加快推进产业转型、创新发展，全力稳增长、促改革、调结构、惠民生、防风险，圆满完成“十二五”规划主要目标任务，全市经济社会保持平稳较快发展的良好态势。

——过去五年，是我市综合实力快速提升、产业结构逐步优化的五年。2011年全市地区生产总值迈过千亿元大关，预计2015年实现1560亿元、年均增长10.8%；规模以上工业增加值年均增长13%，规模以上工业企业利润总额年均增长6.5%、总量保持全省第二位；全社会固定资产投资1280亿元、年均增长19.1%，五年累计完成4702亿元，是“十一五”时期的2.9倍；一般公共预算收入114.6亿元、年均增长15.5%，五年实现翻番；社会消费品零售总额675亿元、年均增长15.5%，实现翻番。预计三次产业结构由2010年的15.4：59.6：25调整为14.5：58.3：27.2，服务业占GDP的比重较2010年提高2.2个百分点。“三个中心、六大基地”规划全面编制实施。战略性新兴产业总产值占全市规模以上工业总产值比重达13%。全市技改投入累计达1126亿元，是“十一五”时期的2.7倍。临港开发区升格为国家级经济技术开发区。成功创建国家农业科技园区，“741”特色优势农业产业格局基本形成。我市被列为川南唯一的全国区域级流通节点城市。

——过去五年，是我市各项事业协调发展、人民生活明显改善的五年。预计全市城镇居民人均可支配收入年均增长12.2%；农村居民人均可支配收入年均增长14.1%；全市累计新增城镇就业人数32.4万人、城镇登记失业率控制在4%以内。社保五大险种全面实现市级统筹，建立了统一的城乡居民基本养老保险制度和医疗保险制度。投入24.2亿元，强力推进农村义务教育“四大工程”建设，翠屏区、南溪区县域义务教育均衡发展顺利通过国家评估认定。深入开展县级公立医院综合改革，实施公共卫生均等化服务。全面落实单独二孩政策。新酒都剧场、市广电中心、市档案馆新馆等大型文化设施相继建成，县（区）图书馆、文化馆等公共文化基础设施不断完善，乡镇综合文化站、农家书屋、社区书屋、广播电视村村通实现全覆盖。社会福利和社会救助体系进一步完善，城乡低保标准逐步提高。成功承办省第十四届民运会，群众性体育活动蓬勃开展，竞技体育成果丰硕。向家坝水电站5.8万移民顺利搬迁。“4·25”双河地震灾后重建全面完成。珙县、筠连县、屏山县新增为少数民族地区待遇县。深入实施扶贫攻坚行动，农村贫困人口累计脱贫60.5万人。成功创建中国杰出绿色生态城市、四川省环境保护模范城市，预计单位GDP能耗累计下降16%，污染物减排任务全面完成。我市成功退出全国二氧化硫和酸雨污染控制区，新创建省级生态县（区）5个，国家级生态乡镇10个、省级47个。全市森林覆盖率由40.7%提高到44.2%，成功创建省级森林城市。南溪实现撤县设区。完成17个乡改镇、3个镇改街道工作，新建农村社区260个。成功创建第三届全国双拥模范城。

——过去五年，是我市重大项目快速推进、城乡面貌焕然一新的五年。宜泸高速建成通车，成贵高铁、宜叙高速等一批重大交通项目开工建设，区域交通枢纽加快形成。宜宾港重大件泊位投入运营，保税物流中心（B型）获批建成，宜宾港获批全国首批进境粮食指定口岸，已建成四川省最大的内河枢纽港。向家坝水电站、珙县电厂一期、福溪电厂一期等一大批产业项目建成投运，建成全国第一口自主经营高产页岩气井、第一条输气管线和全省第一个30万立方米LNG工厂，率先实现页岩气工厂化生产并向居民供气。城市总体规划、历史文化名城保护规划首次通过省政府批准，近50个各组团控规及专项规划编制实施。中心城区和县城品质得到极大提升，累计新增建成区面积88.46平方公里，城镇化率年均提高1.33个百分点，预计2015年达45.9%。成功创建省级园林城市，建成区绿地率达32.8%。小城镇建设成效显著，成功申报全国重点镇12个，省级“百镇建设行动”试点镇18个。

新农村建设迈上新台阶，累计建成千村春风工程示范村2415个、新村聚居点2299个，成片建成新农村省级示范县（片）6个、市级10个。累计新（改）建农村公路8764公里，所有乡镇通油路（水泥路），2831个行政村通水泥路，行政村通畅率达99.6%。

——过去五年，是我市改革开放全面深化、发展活力竞相迸发的五年。制定PPP模式规范化流程，2个项目成功进入国家第一批PPP项目库。五粮液、丝丽雅等国有企业改革有序推进。农业农村体制改革扎实推进，农村产权“七权”同确全面推进，新型农业经营主体和适度规模经营加快发展。市、县（区）政府机构改革顺利完成。事业单位分类工作基本完成。市、县行政许可目录编制工作全面完成。临港开发区改革发展扎实推进。扩权强县、扩权强镇改革加快推进。机关事业单位养老保险制度改革、党政机关公务用车制度改革稳妥推进。教育、科技、文化、卫生体制改革协调推进。生态文明体制改革稳步推进，启动生态文明先行示范区建设试点。民营经济加快发展，民营市场主体由“十一五”末的12.5万户增加到24.1万户，民营经济增加值由464.93亿元增加到910亿元，占GDP比重达58.3%。金融业加快发展，2015年末银行存、贷款余额分别是2010年末的2倍和2.4倍。累计引进金融机构10家，五粮液集团财务公司正式运营。市商业银行第二轮增资扩股全面完成，并设立专营科技支行。在全省率先实现“县级农商行全覆盖”。累计实现招商引资市外到位资金3200亿元，是“十一五”时期的3.3倍；共引进投资项目2351个，引进世界500强、国内100强企业17户。累计实现外贸进出口总额42.16亿美元、引进外资2.47亿美元。与芜湖市等12个国内外城市结为友好城市。大力实施创新驱动战略，新增国家级企业技术中心2个，国家联合地方工程实验室1个、省级自主（重点）实验室4个，省级工程技术研究中心2个。我市第三次蝉联国家科技进步先进市，国家知识产权试点市通过验收，累计获得国家专利授权4835项。县域经济活力显著增强，预计GDP超过百亿的县（区）由“十一五”末的2个增加到8个。

各位代表！刚刚过去的2015年，是宜宾改革发展承上启下重要的一年。面对持续复杂严峻的宏观经济形势，我们认真贯彻落实市委四届九次全会决策部署，按照“稳位调结构、改革求效益”的工作要求，主动适应经济发展新常态，顶住压力、合力攻坚，全面完成了年度目标任务，为我市率先崛起、全面建成小康奠定了坚实基础。

一、突出发展要务，把各级稳增长政策与宜宾实际紧密结合，全市经济保持平稳较快发展。印发《关于抓好省政府稳增长36条措施贯彻落实的通知》，研究出台工业、房地产业、服务业等稳增长75条措施，并确保各项稳增长政策落到实处。全市主要经济指标增速超过或接近全国、全省平均水平，呈现总体平稳、稳中有进、稳中向好的发展态势。预计地区生产总值增长8.5%，总量保持全省第四位；规模以上工业增加值增长9%；一般公共预算收入增长8.5%，总量居全省第三位；社会消费品零售总额增长12.5%，总量保持全省第四位；全社会固定资产投资增长13.2%，总量居全省第五位。

二、突出投资拉动，把项目建设与要素保障紧密结合，项目投资保持快速增长。坚持把稳投资作为稳增长的关键，深入实施项目推进战略，全力推进一批大项目、好项目建设。成贵高铁加快推进，宜叙高速、宜昭高速、绕城高速等项目顺利实施，宜宾机场迁建项目总规获批，川南城际铁路、渝昆铁路、宜攀高速、宜仁高速等项目前期工作顺利推进。金川电子西部磁电产业园、筠连县青蒿素基地等项目投入试运行。新世纪购物广场、西南机械装备贸易城等项目加快推进。三江口长江公园建成投用，南北线出入口改造提升工程取得阶段性成果。人饮安全工程和病险水库整治全面完成，王家沟、新坝、东山、龙滚滩、漂水岩、蟠龙湖等水库加快建设。向家坝灌区北总干渠一期和长江上游干流四川段河堤工程前期工作顺利推进。策划包装拟于2016年实施的投资项目1300余个，总投资近8000亿元。

三、突出民生导向，把发展经济与改善民生紧密结合，民生福祉得到持续改善。预计城镇居民人均可支配收入26354元、增长8.5%；农村居民人均可支配收入11815元、增长10.5%。投入民生工程专项资金107.7亿元，“十大民生工程”及“十七件民生大事”所涉及的138个指标全面完成。社会保险覆盖面不断扩大，各类社会保险参保人数达837万人次。大众创业、万众创新蓬勃开展，建成科技孵化器5个、众创空间30个，发展市场主体29300户、增长44.6%。预计城镇新增就业6.51万人，新增农民工返乡创业1900人，城镇登记失业率3.9%。开工建设保障性安居工程17119套、基本建成15528套、竣工17111套，发放公租房租赁补贴9971户。教育资源布局逐步优化，人民路小学鲁能校区等一批公办学校建成投用，东辰国际学校等一批民办学校入驻宜宾。高考本科上线人数实现“十连增”，2015年达13937人，重本上线人数比上年增长53.2%。四川健康职业学院创建工作有序推进。学前教育、职业教育、高等教育、民族教育、特殊教育、继续教育稳步发展。全面落实国家基本药物制度，基层医疗卫生机构信息化建设快速推进。市二医院临港医院、长江国际医养中心等项目稳步推进。法定传染病发病率连续7年保持全省最低水平。大力实施精准扶贫，创新“五大带动脱贫模式”，构建“六大兜底”保障机制，全年减少贫困人口7.43万人。社会慈善事业稳步发展，养老服务体系逐步完善。开展了纪念抗日战争胜利70周年系列活动。全国质量强市示范城市顺利通过验收。第四届省级文明城市创建工作扎实推进。“平安宜宾”建设全面深化，社会治安综合治理不断加强。食品药品监管工作成效显著。安全生产形势总体稳定，未发生重大及以上事故。信访维稳工作扎实推进，社会保持和谐稳定。国防后备力量持续加强，应急管理体系不断完善，国防教育、人民防空、消防、交通战备扎实推进，民族宗教、新闻出版、电子政务、地方志、档案、公积金、爱国卫生、科协、红十字、工会、青少年、妇女儿童、老龄、残疾人等事业取得新的成绩。

四、突出产业转型，把止滑提速与结构优化紧密结合，产业竞争力不断提升。深入实施工业强市战略，积极推进工业化和信息化深度融合。实施工业企业问题清单制度，“一企一议、一企一策” 帮助企业解决难题。宜宾纸业整体搬迁、中核建中核燃料元件生产线扩建技改工程等48个项目竣工投产。全市净增规模以上工业企业65户，总数达622户。预计“六大基地”企业户数和利润总额分别占到全市规模以上工业的89%、97%，13个重点产业园区累计建成48平方公里，实现规模以上工业总产值1500亿元。五粮液产业园区成功创建国家级新型工业化产业示范基地。农业生产方式加快转变。粮食产量实现“九连增”。“741”特色优势产业快速发展，市级以上农业产业化龙头企业达205户，农村各类专合组织3710个，登记注册家庭农（林、牧）场1964个。建立农业科技试验示范基地45个，建成现代农业万亩示范区15个、万亩林亿元钱示范基地10个、现代畜牧业亿元示范镇（乡）30个。农村电商加快发展，1000余户农特产品企业加入全国知名电商平台。我市被确定为全省唯一的林权抵押贷款改革试点工作整体推进市。现代服务业蓬勃发展，预计服务业增加值增长10.5%。东方时代广场、银龙广场、皇冠假日酒店等项目竣工投运。宜宾欣联物流园等5个项目被列入全省重点物流项目。宜宾港集装箱吞吐量突破20万标箱、增长66%。电子商务园区成功开园，入驻企业36户。成功举办2015四川国际文化旅游节，新增4A级旅游景区3处，荣获中国特色旅游商品金奖3个，签约旅游项目15个、总金额101.1亿元。预计全年实现旅游总收入332亿元、增长29.7%。民生银行宜宾分行、乐山商业银行宜宾分行正式挂牌营业。宜宾保监分局筹建工作顺利推进。

五、突出城乡统筹，把完善设施与加强管理紧密结合，城乡一体化发展加快推进。预计全市完成城乡建设投资360亿元，新增建成区面积17平方公里，城镇化率提高2个百分点。“全域宜宾”规划体系进一步健全，完成33项规划编制工作。临港新区、南部新区、岷江新区、南溪新区、金沙新区、李庄新区、三江口等区域加快开发建设。中心城区城市建设重点项目完成投资105亿元，滨江环线道路工程、旧城立面整治等项目全面完成。环长江旅游景观大道、大溪口至南广镇交通改造及景观工程等项目有序推进。中心城区131条道路黑化及人行道铺装改造竣工投用，拆迁危旧房棚户区32.53万平方米。深入实施城市管理十大治理，中心城区拆除违法建筑9.8万平方米，地下管网、农贸市场、停车场进一步完善。国家卫生城市创建工作通过省级初检。全域数字化城管项目成功创建“全国人居环境范例奖”，国家环境保护模范城市、国家园林城市、国家森林城市创建工作有序推进。全市小城镇建设完成投资15亿元，吸纳转移农业人口3万人。全面实施幸福美丽新村建设“五大行动”，第二轮3个省级新农村示范县、8个市级新农村示范片、千村春风工程三年规划和全省第二轮现代农业、林业、畜牧重点示范县建设任务圆满完成，112个省级财政新村项目完成年度计划，新建成新村聚居点340个、幸福美丽新村451个。

六、突出改革开放，把“引进来”与“走出去”紧密结合，发展动力活力显著增强。着力深化改革，出台《宜宾市重要改革举措实施规划（2015—2020年）》等文件，审议通过专项改革方案27个。大力“引进来”，认真组织参加2015中外知名企业四川行、中国西部（四川）进口展暨国际投资大会，全年实现招商引资市外到位资金828.48亿元、增长14.2%；签约亿元以上产业项目152个，投资677.54亿元。认真做好外资促进工作，嘉士伯重庆啤酒（一期）等项目顺利推进，全年实际利用外资5501万美元、增长8.8%。积极“走出去”，引导企业全面参与“惠民购物全川行动”，成功举办早茶节、中国白酒文化节等系列活动，开展展示展销活动121场（次），实现销售收入492.68亿元。组织99户（次）企业参加“川货全国行”活动，实现销售收入2.1亿元。组织76户（次）企业参加“万企出国门”活动，签订意向性贸易合同7200万美元。加大对五粮液、丝丽雅、惊雷科技、和久集团等重点外贸企业的扶持力度，预计全年外贸进出口总额9.2亿美元、增长4%，实现逆势增长。外事、侨务、台事、贸促、海关、检验检疫等工作成效明显。

各位代表！“十二五”及过去一年取得的成绩，得益于省委省政府和市委的坚强领导，得益于全市上下团结一致、奋力拼搏，凝聚着宜宾565万人民的辛勤汗水和智慧结晶。在此，我代表市人民政府，向各位人大代表、政协委员，并通过你们向全市各族人民、驻宜部队、武警官兵、政法干警、各民主党派、工商联、无党派人士、各人民团体和各界朋友表示衷心的感谢，并致以崇高的敬意！

在肯定成绩的同时，我们也清醒地看到，当前发展中还存在不少困难和问题。受经济下行压力影响，一些企业生产经营仍然困难，全市产业转型、创新发展的压力较大，新的经济增长点还未完全形成；县（区）经济发展不平衡、不协调的现象较为突出，全面深化改革、统筹城乡发展任重道远；社会事业和民生领域欠账较多，脱贫攻坚、生态环保和社会治理的任务还比较繁重。这些困难和问题必须引起我们高度重视，并拿出实实在在的措施加以解决，决不辜负人民的期待和重托！

“十三五”的主要任务

“十三五”时期是全面建成小康社会的决胜期，也是我市经济社会发展大有作为的重要战略机遇期。纵观国际国内形势，我们既有国家推动“一带一路”、长江经济带、成渝经济区建设、全面脱贫攻坚和省委省政府优先发展川南经济区等叠加机遇，同时又面临改革发展深层次矛盾凸显的严峻挑战。我们认为，挑战与机遇并存，机遇大于挑战。只要我们抓住机遇，主动作为，就一定能够实现经济社会平稳较快发展，奋力开创全市改革发展新局面。

根据市委《关于制定宜宾市国民经济和社会发展第十三个五年规划的建议》，市政府起草了《宜宾市国民经济和社会发展第十三个五年规划纲要（草案）》。“十三五”时期发展的指导思想是：高举中国特色社会主义伟大旗帜，全面贯彻党的十八大、十八届三中、四中、五中全会和省委十届七次全会以及市委四届十一次全会精神，坚持以马克思列宁主义、毛泽东思想、邓小平理论、“三个代表”重要思想、科学发展观为指导，深入学习贯彻习近平总书记系列重要讲话精神，紧紧围绕“五位一体”总体布局和“四个全面”战略布局，坚持发展第一要务，牢固树立并切实贯彻创新、协调、绿色、开放、共享的发展理念，以构建绿色宜宾为总体取向，以结构调整、产业转型、创新发展为主线，以提高发展质量和效益为中心，深入实施“2365”发展战略，推动新型工业化、信息化、城镇化、农业现代化同步发展，加快形成引领经济发展新常态的体制机制和发展方式，统筹推进经济、政治、文化、社会和生态文明建设，确保在全省次级突破战略布局中率先崛起、先于全省全面建成小康社会。

“十三五”时期我市发展的主要目标是：全市地区生产总值年均增长8%以上，到2018年地区生产总值和城乡居民收入比2010年翻一番；到2020年地区生产总值达到2300亿元、人均地区生产总值接近全省平均水平并与全国差距进一步缩小，加快建成经济总量大、经济结构优、创新能力强、质量效益好的经济强市。经济发展的质量和效益显著提升，产业转型、创新发展迈出实质性步伐，绿色宜宾建设取得明显成效，人民生活水平达到小康，基本建成长江上游辐射吸纳川滇黔的区域中心城市，如期实现率先崛起、全面建成小康的奋斗目标。

——坚持创新发展，着力培育新的发展引擎。树立创新发展理念，重要领域和关键环节的改革取得实质性突破，加快形成促进创新的体制框架。加快推进科技创新，引导和支持企业建立国家级企业技术中心2个、工程技术研究中心5个，国家级和省级重点实验室4个。建立集人才、资源、大数据为基础的综合服务平台，实施一批重大产学研合作和重大成果转化项目，“十三五”末战略性新兴产业总产值占全市规模以上工业总产值的比重达到18%以上。创建科技孵化器10个、众创空间100个。推动一二三产业融合发展，“三个中心、六大基地”主营业务收入突破3000亿元，绿色食品工业城市加快建设，服务业发展质量显著提升，三次产业结构调整到10：58：32左右。

——坚持协调发展，切实增强发展的整体性。树立协调发展理念，坚持区域协同、城乡一体、物质文明精神文明并重，促进“四化”同步发展。推动区域协调发展，形成以中心城区为核心、以县城和重点城镇为主的城镇发展格局，中心城区新增建成区面积45平方公里以上，建成幸福美丽新村2270个以上。加快打造沿江经济带，加大山区综合开发力度，做强做大县域经济。统筹城乡一体化发展，进一步完善城镇基础设施和公共服务，实现城乡要素平等交换、合理配置和基本公共服务均等化，到2020年城镇化率达55%。推动物质文明和精神文明协调发展，现代公共文化服务体系基本建成，公民文明素质显著提高。

——坚持绿色发展，加快推进绿色宜宾建设。树立绿色发展理念，创新绿色发展模式，生态、产业、城市、文化绿色化发展水平明显提高，单位GDP能耗完成省上下达目标任务。人居环境明显改善，城市建成区绿地率达到35%以上。实行最严格的环境保护制度，主要污染物排放总量控制在合理水平，资源综合利用水平显著提高。生态环境质量持续改善，所有县（区）达到生态县建设标准，累计建成国家级、省级生态乡镇100个，全市森林覆盖率达到45%以上，加快构建长江上游绿色生态屏障。

——坚持开放发展，不断丰富对外开放内涵。树立开放发展理念，市场在资源配置中的决定性作用得到充分发挥，开放型经济新体制基本形成。加快构建“四高七普一枢纽”11条铁路干线网、“一绕九射”12条高速公路骨架网和“五横五纵”快速公路网，加快推进长江、岷江、金沙江航道等级提升工程，完成宜宾新机场迁建，开放合作条件全面优化。招商引资取得显著成效，力争实现招商引资市外到位资金4500亿元以上，引进世界500强、国内100强和行业10强企业15户以上。深化与国内外区域交流合作，提升贸易便利化水平。

——坚持共享发展，促进发展成果全民共享。树立共享发展理念，坚持普惠性、保基本、均等化、可持续方向，统筹抓好义务教育、就业服务、社会保障、基本医疗和公共卫生、公共文化等各项事业改革发展。实施更加积极的就业创业政策，努力提高城乡居民收入。加快普及学前教育和高中阶段教育。食品安全等公共安全更有保障，农产品质量安全例行监测总体合格率达到96%以上。坚决打赢脱贫攻坚战，到2019年全面完成农村贫困人口脱贫，贫困县、贫困村全面摘帽，消除绝对贫困。积极应对人口老龄化，促进人口长期均衡发展。大力发展文化事业和文化产业，加快建设文化强市。依法治市全面落实，社会治理体系和治理能力现代化取得重大进展。

各位代表，以上目标任务，既做到了与率先崛起、全面建成小康的目标相衔接，又充分考虑了未来五年发展的条件和趋势，是一个客观积极的目标，符合经济发展规律、符合宜宾发展实际。特别是保持适当高于全省的经济增长速度，这是宜宾未来发展的底线，是创新发展、转型发展、绿色发展的根本要求和必然结果，也是全市人民的新期盼。同时，考虑到经济进入新常态，我们面临速度换挡、结构优化、动力转换等现实情况，实体经济运行还面临不少的困难，经济很难在高位上持续高速增长，确保一个合理的增长速度，可以为转方式、调结构留出回旋空间，为防范经济风险留有余地。各县（区）要从实际出发，抢抓机遇、积极进取，努力争取更好结果，有条件的可以竞相跨越、加快发展。我们坚信，只要全市上下紧扣目标、共同努力，就一定能够实现经济社会发展新跨越，谱写“四个全面”宜宾实践新篇章！

2016年工作安排

2016年是实施“十三五”规划的开局之年，做好今年的工作十分关键。我们要认真贯彻落实党中央国务院、省委省政府和市委的一系列决策部署，按照“稳定增长调结构、改革创新促转型”的总要求，始终保持创新发展、转型发展、绿色发展定力，以建设绿色宜宾为总体取向，大力实施“2365”发展战略，着力加强结构性改革，在适度扩大总需求的同时，正确把握用好中央“去产能、去库存、去杠杆、降成本、补短板”的重大机遇，提高投资有效性，提高供给体系质量和效率，加快培育新的发展动能，改造提升传统比较优势，推动全市社会生产力水平整体改善，努力实现“十三五”发展良好开局。

今年全市经济社会发展的主要预期目标是：地区生产总值增长8%以上；规模以上工业增加值增长8.5%以上；服务业增加值增长9.5%以上；全社会固定资产投资增长10%以上；社会消费品零售总额增长11.5%以上；一般公共预算收入增长8%以上；城镇化率提高2个百分点以上；城镇新增就业5.5万人以上，城镇登记失业率控制在4.5%以内；城镇居民人均可支配收入增长8%以上；农村居民人均可支配收入增长9%以上；完成省上下达的物价调控、节能减排等目标任务。

围绕今年确定的预期目标，我们要着力抓好八个方面的重点工作。

一、着力稳定经济增长，加快培育新的经济增量

切实向项目投资要增量。大力推进“项目年”行动计划，围绕“三个中心、六大基地”建设，着力优化投资结构，保持投资持续快速增长。突出抓好72个省重点项目和196个市重点项目建设，加快推动成贵高铁、宜宾机场迁建、宜叙高速、宜昭高速、绕城高速及环长江旅游景观大道等重点项目建设，推动川南城际铁路、宜威高速、宜仁高速、长江航道宜宾段“三升二”等重大项目尽快开工，积极做好渝昆铁路、宜西铁路、宜攀高速、金沙江宜宾至水富段三级航道整治等项目前期工作。加大页岩气资源开发利用力度。全力推进149个2000万元以上重点技改项目建设，确保工业投资375亿元以上。加快推进向家坝灌区北总干渠一期工程、长江上游干流四川段河堤工程、6个中型水库、3个烟水工程等一批骨干水利项目建设。

切实向扩大消费要增量。全力抓好“惠民购物全川行动”“川货全国行”“万企出国门”三大活动，开展好迎春购物月、早茶节、采购商大会、中国白酒文化节等一批全市性重大活动。大力促进信息消费、健康养老消费、节假日消费，积极培育市场消费热点。扎实抓好电子商务产业园建设发展，培育和引进一批有影响力的电商企业，积极创建国家电子商务示范城市。大力发展网购网销，鼓励企业在全国区域中心城市开设营销网点和“形象店”，着力提升“宜宾造”产品的影响力和知名度。

切实向壮大市场主体要增量。按照“企业+资本+互联网+科技创新”的思路，推动市场主体做大做强。加快实施五粮液集团“千亿工程”，丝丽雅集团和天原集团“三百亿工程”，中核建中、惊雷科技和宜宾纸业 “百亿工程”。积极支持“补链”“强链”的中小微企业发展，大力推进“个转企” “企升规”和“规改股”，全年新增规模以上工业企业50户以上。支持有条件的企业通过兼并重组优化资源配置、完善重点产业链、扩大规模效益、提升创新能力。

切实向做大做强民营经济要增量。着力营造宽松平等的准入环境，鼓励和支持各种所有制企业创新发展，保护其产权和合法利益。进一步放宽社会资本进入基础设施、公用事业等行业和领域的限制。加强对民营企业在资金、土地、政策等方面的支持，鼓励和引导民营企业建立现代企业制度，促进民营经济持续健康发展。

切实向推动新型城镇化建设要增量。积极争取地下综合管廊、海绵城市、智慧城市、棚户区改造等公共基础设施建设的政策支持，进一步加快新型城镇化进程。着力推进城乡统筹发展，切实加大城市改造提升、城镇功能完善、小城镇建设、农村基础设施和公共服务设施建设投入，不断挖掘投资方向和消费需求。

切实向开放合作要增量。用好中外知名企业四川行等投资促进平台，综合用好小分队招商、资源招商、产业招商、项目招商、以商招商等方式，力争全年招商引资到位资金880亿元以上。抓好已签约项目的落地，确保项目早开工、早见效、早达产。加快推进川南经济区一体化发展，深化与长三角、珠三角、环渤海等区域合作，加强与法国、德国、俄罗斯、韩国、东盟等国家和地区的交流合作。引导和鼓励重点外贸企业扩大对“一带一路”沿线国家的贸易和投资，支持白酒、茶叶、丝绸、竹制品等特色产品出口，力争全年外贸进出口总额达到9.5亿美元以上。

二、着力加快结构调整，推进产业转型升级

加快推进工业经济转型。主动对接“中国制造2025”“互联网+”行动计划，紧扣“六大基地”建设推动产业转型升级，加快构建绿色工业体系。更加注重“宜宾酒”品牌打造和市场占有率的提高，进一步完善扶持白酒产业发展的相关政策，促进产品结构调整和市场营销。更加注重新能源的开发和就地转化利用，加快转变煤炭生产经营模式，力争开工建设筠连2×35万千瓦煤矸石发电项目。更加注重高端装备制造和智能制造，推动装备制造产业和重点企业快速发展。更加注重行业地位和产品市场占有率的提升，促进化工轻纺建材产业结构优化升级。更加注重绿色食品工业城市的打造，加快建设绿色食品精深加工基地。更加注重新型化工、生物医药、电子信息、新材料、文化创意等新兴产业培育发展。加快13个重点产业园区建设，打造高端工业创新平台，鼓励和支持符合条件的园区创建省级经济技术开发区、高新技术产业开发区。加快长江工业园区规划建设。

促进现代服务业快速健康发展。完成《宜宾市“十三五”服务业发展规划》编制，加快全市服务业聚集区建设，大力发展与现代制造业配套的现代物流、现代金融、商务服务等生产性服务业，积极发展卫生、文化、体育以及家政、养老、快递等生活性服务业，加快培育创意设计、商务会展、物联网等新兴服务业。加快推进新世纪购物广场、西南机械装备贸易城等重大项目建设，优化城市大型商业综合体、购物中心、农贸市场等网点结构和布局。加快象鼻物流园区、菜坝配送中心等物流节点建设，加强农产品冷链物流扶持，推进农村物流配送体系建设。大力发展现代旅游业，启动国家旅游标准化示范市创建工作，抓好旅游产品体系和市场营销体系建设，拓展国际国内市场，力争全年实现旅游总收入400亿元以上。加快蜀南竹海、兴文石海、五粮液、李庄古镇等景区建设，积极创建国家5A级景区、国家级旅游度假区、国家生态旅游示范区等品牌。推动旅游与文化、农林、工业、科技融合发展，大力发展生态文化旅游、休闲度假旅游、乡村旅游、红色旅游和自驾游。围绕吃住行游购娱等旅游要素配套升级，打造“好吃在宜宾”的饮食文化品牌和演艺、体验产品，开发提升旅游商品和购物基地。培育壮大现代金融业，扎实抓好成都农商行入驻、组建宜宾市农商银行等工作，鼓励银行、保险公司等金融机构在县（区）设立分支机构。做好“创业板行动计划”入库工作。

加快绿色食品工业城市建设促进现代农业发展。按照“立足现有农产品加工业提升一批、依托科研院所科技成果孵化转化一批、瞄准市场影响力大的龙头企业招大引强一批、紧盯产业同构企业嫁接一批、围绕构建农业全产业链延伸一批”的要求，加快培育适应不同年龄、不同职业、不同人群需求的休闲食品、保健食品、绿色食品，打造绿色食品品牌。现代农业发展要在特色上下功夫，大力发展“741”特色优势产业，推进中低产田改造和高标准农田建设，创建一批现代农业产业化示范基地，建设一批优质农产品生产基地和集配中心。采取财政资金奖补等措施，扶持发展多种形式的农业适度规模经营。加快推进国家农业科技园区基础设施建设，积极组建绿色食品产业技术研究院和科技孵化器，不断增强园区的研发、生产、生态、孵化培育、休闲观光等功能。强力推进农村电商发展，促进农产品进城和工业品下乡双向互动。强化农产品质量安全，抓好“三品一标”农产品认证登记保护工作，着力打造宜宾农业品牌。

三、着力推进改革创新，加快培育经济增长新动能

全面深化改革。深化投融资体制改革，构建财政资金和融资平台良性互动机制，用好用活国资公司等投融资平台，引导金融机构加大对转型升级企业和项目的信贷支持力度。推广政府和社会资本合作、特许经营、政府购买服务等方式，充分激活民间投资。深化商事制度改革，进一步精简行政许可事项，推进“三证合一”“先照后证”改革，促进工商登记便利化。深化国资国企改革，积极发展混合所有制经济，下大力气抓好五粮液、丝丽雅、天原、宜宾纸业等重点企业改革发展。深化农村产权制度改革，积极推进林权、土地经营权、农村房屋产权、动物活体抵押贷款以及农村资金互助试点等工作，按期完成土地承包经营权确权颁证，依法推动土地经营权有序流转，积极发展多种形式的适度规模经营。稳步推进农村集体资产股份合作制改革，有序推进供销社改革。积极推进基本养老保险制度、医药卫生体制、户籍制度等重点领域改革，完成党政机关公务用车制度改革。

深入推进创新发展。鼓励企业充分发挥资源、资本和技术优势，开展基础性、前沿性创新研究，突破一批具有自主知识产权的核心技术、关键技术和共享技术，力争技术创新投资增长20%以上。加大对中小微企业创新能力建设的支持力度，鼓励开展产品创新、技术创新、运营模式创新。大力实施创新主体培育工程，支持企业联合高校院所共建产业技术研究院、产业（技术）创新联盟，力争全年创建高新技术企业10户。加大知识产权保护力度，促进科技成果就地转化，积极争创国家创新型城市和国家知识产权示范城市。实施“互联网+”行动计划，推进产业组织、商业模式、供应链、物流链创新，促进互联网和经济社会融合发展。积极推动大数据广泛深度应用，推进大数据开放共享、云技术创新发展，加快建设智慧城市。

加快临港开发区发展。积极推进以市场化为导向的实体化团队制度改革，创新创优开发建设模式。强化先进装备制造业、新能源汽车等主导产业培育，构建现代产业体系。加快散货泊位、进港铁路、盐坪坝长江大桥、临港国际物流园区、国别园区等重大项目规划建设，尽快形成完善的集疏运体系。全力推进长江干流起点航运物流中心建设，加强宜宾港与上海港、武汉港、重庆港等港口合作，大力发展公水、铁水等多式联运，力争集装箱吞吐量达到23万标箱以上。

推进大众创业、万众创新。建立完善创新创业扶持政策和服务体系。建立健全大学生创新创业导师制，推进大学生创新创业俱乐部和创新创业园建设。大力开展群众性创新创业活动，鼓励行业领军企业、创业投资机构、社会组织积极参与或自主建设众创空间，支持农民工、农民企业家等返乡创业。加快打造创新创业合作平台，建设一批创新创业园（孵化基地），逐步形成阶梯型孵化体系。大力推行众创、众扶、众包、众筹等新模式，打造科技孵化器5个、众创空间50个。

加强企业家队伍建设。树牢“像尊重科学家、尊重老师一样尊重企业家”的理念，向企业家学习，尊重企业家的创新创业精神。加大对企业家的宣传力度，在全社会形成尊重企业家、尊重企业创造的氛围。严格实行企业安静生产制度，为企业发展创造和谐环境。严厉打击各类扰乱企业的行为，坚决纠正简单粗暴执法，创造公正公平的法治环境。加强企业家队伍培训，着力提高创新创业能力和经营管理能力，不断壮大企业家队伍。

四、着力推进供给侧改革，切实破解发展瓶颈

提高要素供给效率。继续打好支持实体经济发展的政策措施组合拳，着重从企业用水、用电、用气、物流、融资等生产要素全方位谋划，打造产业发展政策洼地。积极争取用地计划指标，清理闲置土地、盘活存量土地，保障项目建设用地需求。认真落实金融支持实体经济的政策措施，引导金融机构加大对实体经济特别是小微企业的支持力度。做好经济运行监测预警工作，有针对性地解决企业发展中的困难和问题。

切实减轻企业负担。开展降低实体经济企业成本行动，严格清理和规范涉企收费，降低企业物流运输等要素成本，进一步清理规范中介服务。积极争取和用好直购电、丰水期富余电消纳等电力政策，争取天然气直供和页岩气就地就近低价利用政策支持。稳妥处置“僵尸企业”，消除“空壳公司”，妥善解决人员安置、债务负担等问题，防范引发社会风险。

积极化解房地产库存。通过户籍制度、土地制度等改革，提高户籍人口城镇化率，加快农民工市民化。深化住房制度改革，提高住房公积金使用率，积极发展住房租赁市场，提供有效住房供给，满足新市民住房需求。高度重视商品房库存、空置问题，摸清底数，及早谋划应对措施。科学调控土地出让量及房地产开发量，积极培育本地建筑企业，促进房地产业健康、有序、多元发展。

有效防范金融风险。依法解决涉众性民间借贷和非法集资问题，健全担保体系，完善金融监管机制，坚决守住不发生系统性和区域性金融风险的底线。着力防范和化解地方政府性债务风险，加强债务监测监管，规范举债行为，积极争取国家债务置换，推动地方政府融资平台市场化转型，保持债务风险在可控范围之内。

五、着力实施精准扶贫，打好扶贫开发攻坚战

解决好“扶持谁”的问题。按照“科学制定规划、措施精准到户、着力造血扶贫、政策扶持兜底、防止脱贫反弹、强化主体担当”的扶贫工作总体取向和“脱贫是根本要求、不返贫是目标要求、达小康是最终要求”的工作任务，科学编制全市“十三五”扶贫开发规划。建立扶贫信息系统数据对接共享机制和贫困动态管理机制，制定扶贫攻坚项目台账，因户施策、因人施策，确保全年实现90个贫困村摘帽、6.66万人脱贫。切实推进移民后扶发展，健全移民安置区交通、水电气等基础设施，加快完善教育、医疗卫生等服务体系。

解决好“谁来扶”的问题。突出“六个精准” 要求，层层签订脱贫攻坚责任书，精准落实扶贫责任。建立扶贫攻坚督查调研机制和巡回督导制度，健全驻村干部管理办法，加强督查问责。建立社会资金投入扶贫开发引导机制，鼓励支持各类企业、社会组织、个人参与扶贫攻坚，加快形成全社会参与的大扶贫工作格局。建立贫困帮扶投诉举报机制，加强扶贫项目资金的监督管理，严厉打击侵害贫困户利益的违纪违规行为。

解决好“怎么扶”的问题。认真实施“五个一批”行动计划、“六大兜底”保障机制、“十个扶贫专项方案”和年度工作计划，加快完善贫困村脱贫规划、贫困户帮扶计划，统筹解决好插花贫困问题。切实加强产业培育和就业帮扶，提高贫困地区“造血”能力。有效整合各类扶贫资源，加大资金、政策投入力度，探索把住房改善、基础设施、产业发展、公共服务等涉农项目打捆使用。加大对贫困地区转移支付补助力度，鼓励引导各类金融机构加大对贫困县、贫困村、贫困户的金融支持。健全城乡居民大病救助机制和大病保险制度，努力防止因病致贫、因病返贫。

六、着力推动城乡统筹，加快城乡一体化发展

加快推进新型城镇化进程。不断完善“全域宜宾”规划体系，科学规划城市空间布局。加快推进临港新区、南部新区、岷江新区、金沙新区、李庄新区、南溪新区等区域开发建设，力争完成城乡建设投资365亿元，新增建成区面积16平方公里。扎实开展“城市基础设施建设年行动”，加速推进三江口区域综合开发、大溪口至南广镇交通改造及景观工程、南溪长江公路大桥、城市危旧房棚户区改造等项目建设。强化中心城区各组团与县城之间的产业协作协同，逐步形成横向错位发展、纵向分工协作的发展格局。切实抓好全国重点镇、“百镇建设行动”试点镇、市级重点镇和试点镇建设工作。

提升城市管理和服务水平。大力推进绿色城镇化，积极推进生态城市建设，努力创建国家园林城市、省级文明城市。确保成功创建国家卫生城市。加强城市精细化管理和服务，完善“大城管”体制机制，着力解决城市病等突出问题。统筹抓好交通秩序、标识标牌、园林绿化等城市管理十大治理。加大公共交通工具投放力度，合理规划布局公交线路、站台和红绿灯等交通基础设施，新建公交车停车场4个。

加快发展县域经济。加强县域交通、水利、能源、信息等基础设施建设，改善县域经济发展条件。深化扩权强县、扩权强镇改革，完善县级财政税收体制，扩大县域经济发展的自主权。健全县域产业合作发展和利益分享机制，鼓励县（区）共建产业合作园区，共同培育区域公共品牌和地理标志保护产品。推进跨区域基础设施互联互通、产业对接协作、公共服务共建共享和环境联防联治。有序推进“撤县设市”和“撤乡设镇”工作。加大对贫困地区、民族地区、革命老区的政策、项目和资金支持。

切实推进幸福美丽新村建设。全面实施幸福美丽新村建设“五大行动”，推动“物的新农村建设”向“人的新农村建设”迈进。加强资金整合和项目统筹，扎实抓好第三轮新农村成片推进示范县和现代农业、林业、畜牧示范县建设，启动4个省级新农村示范县、6个市级新农村示范片建设。扎实推进乌蒙新村建设，全年建成幸福美丽新村450个。新建农村社区20个。

七、着力推动绿色发展，加快建设绿色宜宾

大力发展绿色经济。按照市委《关于加快建设绿色宜宾的决定》，抓紧编制绿色宜宾规划和十大绿色行动计划的实施方案。加快传统制造业绿色改造，推行企业循环式生产、产业循环式组合、园区循环式改造，减少单位产出物质消耗。积极建立绿色发展基金。推广农业清洁生产技术，强化品种选择和投入品控制，积极发展有机农业、生态农业，因地制宜发展特色经济林、林下经济和森林旅游等产业。加快实施服务业标准化试点项目，大力推动商贸物流、金融、旅游等产业绿色发展。加速发展绿色能源产业，加快建设国家级页岩气创新开发示范区，积极推广充换电基础设施、生物质燃气、生物质液体燃料应用。探索建立环保第三方治理机制，加快环保产业发展。

切实推进生态文明建设。建立绿色宜宾指标评价体系、考核办法、奖惩机制，完善生态文明建设和环境保护实绩考核制度。深入推进退耕还林、生物多样性保护等工作，建立天然林总量管理制度，增加森林面积和积蓄量。加快创建国家森林城市。实施山水林田湖生态保护和修复工程。编制实施长江、金沙江、岷江水质达标规划及南广河、越溪河等重点小河流域治理保护规划。全面推进向家坝库区等重点区域水土流失、南部区域石漠化综合治理，加快建设长江上游绿色生态屏障。

狠抓节能减排工作。实行环境质量和污染排放总量双控，加强污染物排放浓度、速率、总量的达标常态化减排管理。严控高耗能行业新增产能，积极化解过剩产能、淘汰落后产能。严格项目准入门槛，积极促进节能环保企业和项目投资落户我市。积极倡导绿色低碳的生活方式和消费行为，大力推广使用新能源汽车、高效节能系列产品。

加大环境保护力度。深入实施大气污染防治行动计划，重点控制PM2.5、PM10、挥发性有机物等大气污染物。深入实施水污染防治行动计划，落实严格的饮用水源保护措施，大力整治“三江九河”沿线水污染源。完善城市空气质量、重点流域水质自动监测网络体系建设。推进土壤污染防治，加大规模化畜禽养殖污染防治力度。强化工业固体废物、重金属等污染防治，建立健全化学品、持久性有机污染物、危险废物等环境风险防范与应急管理工作机制。加快工业集中区污水收集处理，推进城镇生活污水、生活垃圾处理设施建设。积极创建国家环境保护模范城市。

八、着力保障和改善民生，促进社会和谐稳定

加强就业和社会保障工作。积极抓好返乡农民工、高校毕业生、退伍军人等重点群体就业工作，强化职业技能培训。完成城乡居民医保管理制度整合。稳步推行居民健康卡、川南通、市民卡，加快“三基一卡”和“社银平台”建设力度，社会保障卡持卡人数达到430万人以上。加大“五保”“低保”和城市“三无”人员等特殊困难群体救助帮扶力度。大力推进保障性安居工程建设，新开工保障性安居工程8950套、基本建成14000套，新增发放公租房租赁补贴435户。

协调推进教育、卫生、文化、体育等社会事业发展。全面深化教育综合改革，不断优化教育资源布局，提升教育教学质量。全面完成农村义务教育“四大工程”配套设施、省市“改薄工程”建设任务，推进义务教育均衡发展。加快推进教育信息化建设。提高学前教育普及程度。加快发展中职教育，推进高中阶段教育普及。大力发展民办教育，促进宜宾东辰国际学校、四川师范大学附属实验学校等重点项目建设。全力抓好四川健康职业学院创建工作，积极推进宜宾职业技术学院职业教育综合改革，推动宜宾学院向应用型综合大学转型发展。加快建设“健康宜宾”，深化县级公立医院综合改革，巩固基本药物制度成果，推进异地就医结算管理。全面提升基层卫生服务能力，推广“县乡村”一体化改革，推行“县招乡用、乡招村用”的创新模式。积极实施分级诊疗制度，强化重点疾病防治，提高公共卫生事件应急处置能力。加快推进二医院临港医院、妇幼保健院迁建、长江国际医养中心等项目建设。全面实施一对夫妻可生育两个孩子政策。深化文化体制改革，大力发展文化产业、规范文化市场、加强文艺创作，加快构建现代公共文化服务体系。积极推进传统媒体与新兴媒体融合发展。扎实推进市规划馆、市博物馆等重点项目建设。大力开展全民健身运动，举办市第五届运动会，启动市体育活动中心项目建设。积极协调推进宜宾军供站迁建前期工作。深入开展“全国敬老文明号”创建活动。抓好第三次全国农业普查工作。继续抓好民族宗教、地方志、爱国卫生、红十字、电子政务、科协、档案、工会、青少年、妇女儿童、老龄、残疾人等事业发展。

创新社会治理方式。深化“平安宜宾”建设，健全社会治理体系、公共安全体系和立体化社会治安综合防控体系，加强流动人员管理，严厉打击各类违法犯罪活动，强化反恐防暴斗争。深入推进“法律七进”，深化法治示范创建，加强全民普法教育。深化社会稳定风险评估，健全利益平衡和多元化矛盾纠纷调处体系，引导群众依法反映诉求，依法处理涉法涉诉信访问题。深化特殊人群服务管理综合改革试点，完善城乡社区网格化服务管理体系。加强社会信用体系建设。深化社会组织管理改革，健全完善政社分开长效机制。切实构建综合监管、行业监管、专业监管互补配合的安全监管体制，扎实开展安全生产隐患排查整治。强化食品药品安全监管。加强以消防为骨干的多种形式应急救援力量建设。做好地震、测绘、气象、水文工作。加强国家安全和保密工作。

加强国防建设。积极盘活军地科技、教育、人才、信息等资源，促进军民融合深度发展。积极支持国防和军队改革。深入开展双拥模范城（县、区）创建活动。切实做好国防教育、国防动员、人民防空、交通战备、优抚安置等工作，积极抓好征兵和民兵预备役建设。

加强法治政府建设

各位代表，“十二五”时期，我们大力推进政府自身建设，各项工作都取得了较好成效。我们坚持依法行政，认真落实市人大及其常委会的决议决定，认真听取各民主党派、工商联和无党派人士的意见，累计办理人大代表建议1834件、议案8件和政协委员提案1396件、意见39件，办复率为100%。我市被列入全省健全完善政府法律顾问制度改革试点地区，获得第三届中国法治政府奖提名奖、第八届中国政府创新优秀实践奖。我们坚持提升效能，积极推进并联审批和三级联动审批，行政权力依法规范运行平台和监察平台全面运行。我市被确定为全国第一个地（市）级的国家电子招投标试点城市。我们坚持改进作风，积极开展党的群众路线教育实践活动和“三严三实”专题教育，切实加强重大问题调研和政府目标绩效管理，2015年政府系统全市性会议比2010年减少18.9%。深入实施环境兴市战略，持续开展“庸懒散浮拖”专项整治。着力加强12345市民服务平台建设，群众总满意率达99.8%。我们坚持正风肃纪，着力解决违反中央八项规定精神和发生在群众身边的腐败问题，累计查处案件530件，挽回和避免经济损失1.14亿元。

同时，我们也深刻认识到，与发展的要求和群众的期盼相比，政府自身建设还有一定差距。我们将进一步优化政府服务，不断提升行政效能，奋力推动法治政府建设再上新台阶！

强化依法行政，打造法治政府。认真贯彻党中央、国务院《法治政府建设实施纲要（2015—2020年）》。落实依法行政主体责任，严格按照法定权限和程序履行职责、行使权力，主动接受党内监督、人大的法律监督和工作监督，自觉接受政协和民主党派监督以及司法监督、社会监督、舆论监督。强化行政监察和审计监督。认真落实政府常务会议和部门办公会议学法制度，坚持重大行政决策法定程序，不断提高运用法治思维和法治方式深化改革、推动发展、化解矛盾、维护稳定的能力。

强化环境兴市，打造服务政府。深化环境兴市战略，持续推进行政审批改革，扎实推进履职行权规范化建设。深入推进政务公开。加快打造实体服务大厅和网上办事大厅线上线下一体的政务服务平台，真正做到“一窗式受理、一站式审批、一条龙服务”。畅通环境兴市“二维码”测评、正风肃纪“随手拍”等渠道，引导企业和群众参与到环境建设中来。进一步优化提升12345市民服务平台功能，加快打造全国一流服务平台。

强化作风建设，打造效能政府。坚持“严”的精神和“实”的作风，强化政府班子建设，加强干部教育管理。深化政府目标绩效管理，探索建立市政府重要文件贯彻落实效果评价机制，坚决处理懒政、庸政、怠政等消极现象，全面落实首问责任制、限时办结制和责任追究制，不断提升机关效能。持续推进作风转变，将中央八项规定、省委省政府和市委十项规定精神落实到政府工作的方方面面。加强重大问题调研分析。切实转变会风、文风。

强化廉政建设，打造廉洁政府。坚决贯彻从严治党、从严治政各项规定，认真落实党风廉政建设责任制。扎实推进政府系统惩治和预防腐败体系建设。继续严控“三公”经费和会议费。积极推进对所有公共资金、国有资产、国有资源和领导干部履行经济责任情况的审计监督全覆盖。深化正风肃纪专项治理，纠正行业不正之风。严肃查处腐败案件，始终保持惩治腐败的高压态势。

各位代表！让我们紧密地团结在以习近平同志为总书记的党中央周围，在省委省政府和市委的坚强领导下，团结带领全市各族人民，进一步解放思想，改革创新，求真务实，锐意进取，为加快实现率先崛起、全面建成小康宏伟目标而努力奋斗！
